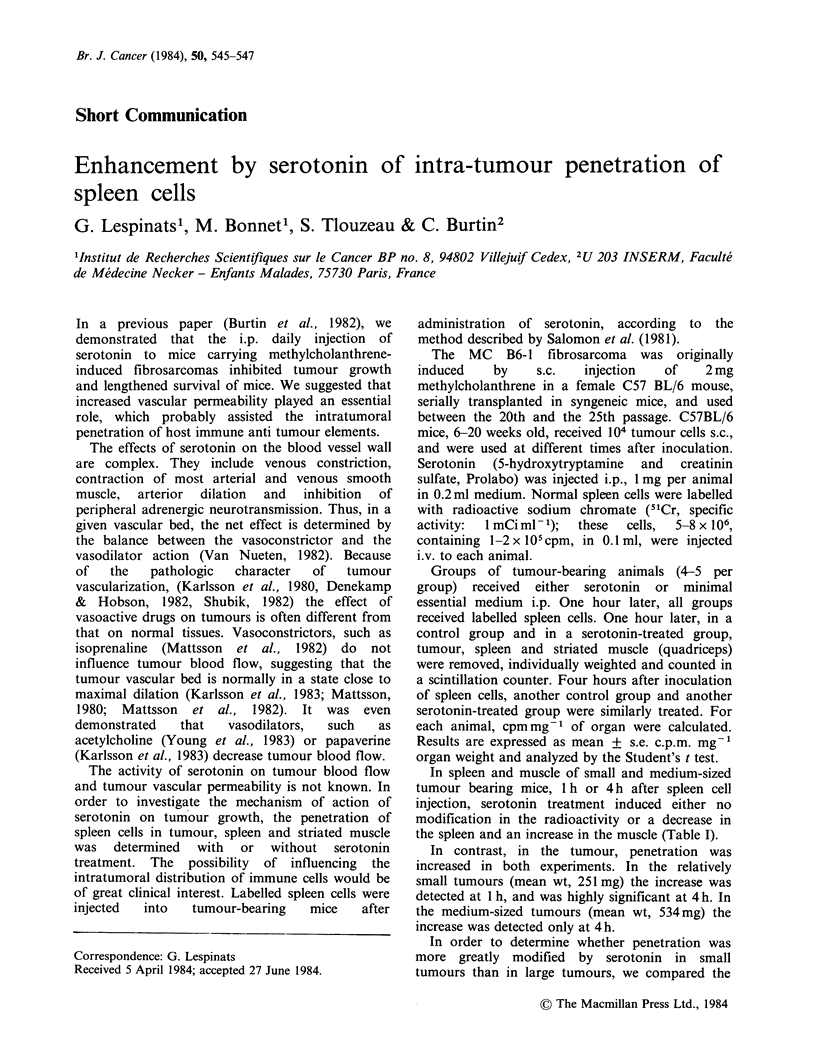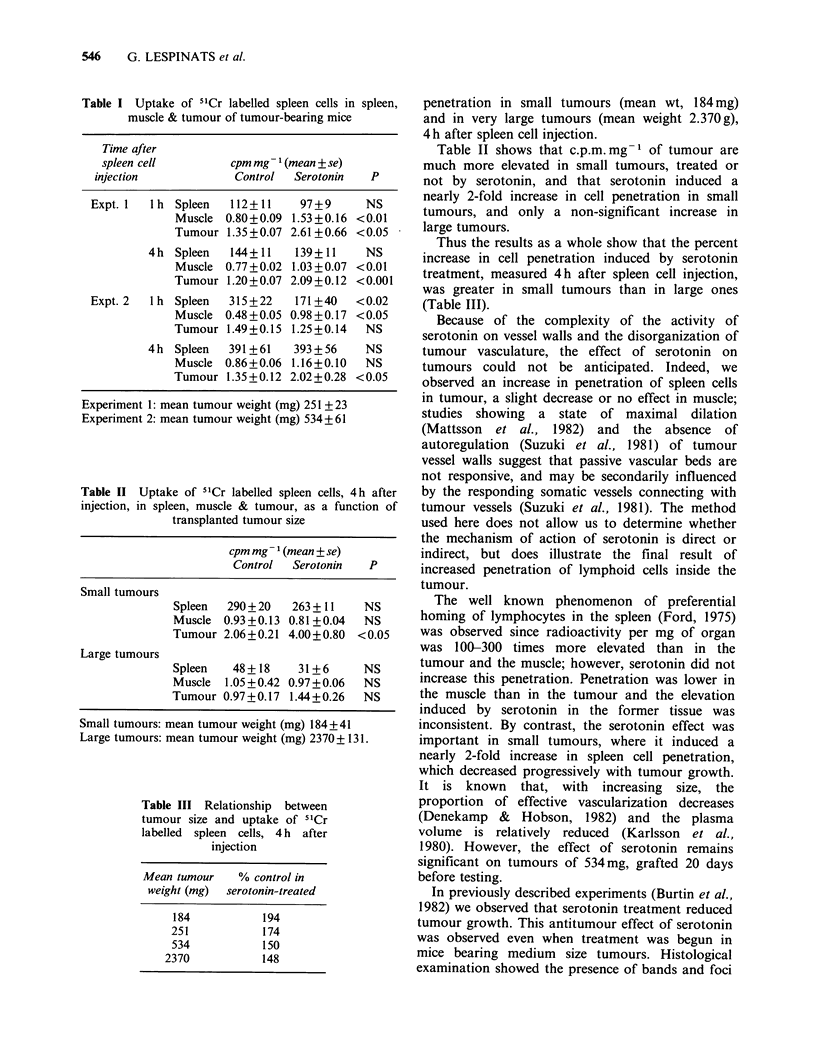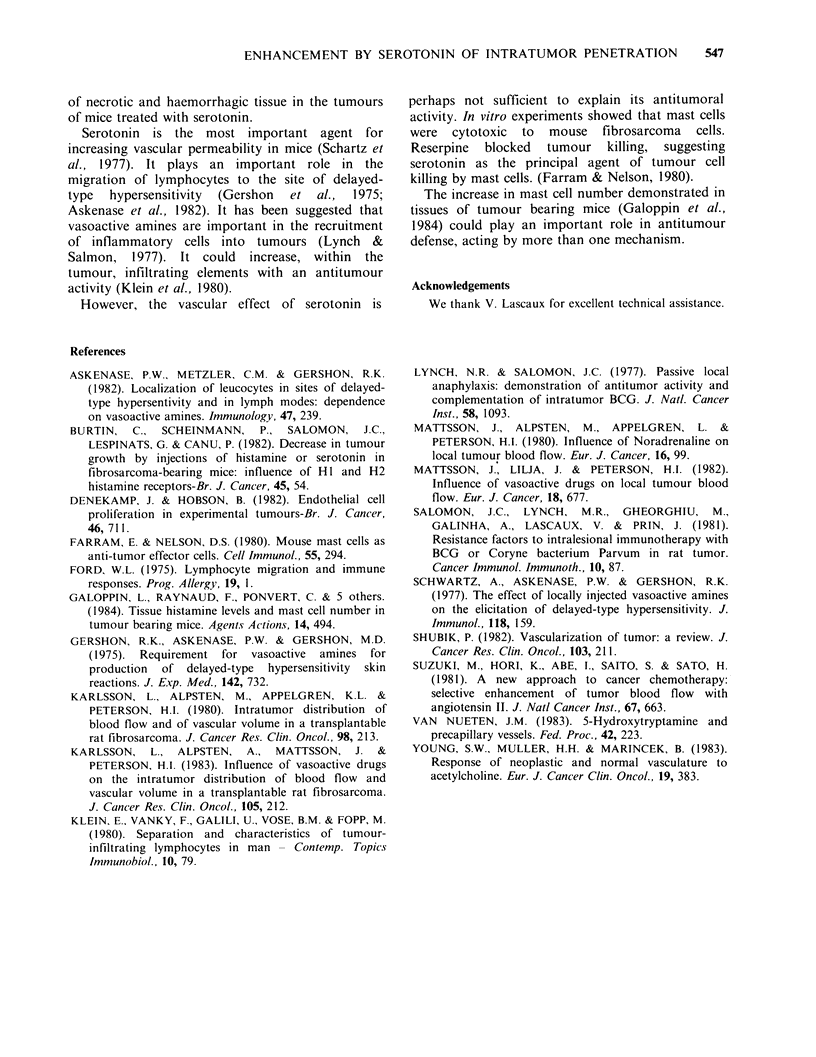# Enhancement by serotonin of intra-tumour penetration of spleen cells.

**DOI:** 10.1038/bjc.1984.214

**Published:** 1984-10

**Authors:** G. Lespinats, M. Bonnet, S. Tlouzeau, C. Burtin


*Br. J. Cancer* (1984), **50**, 545–547

### Short Communication

### Enhancement by serotonin of intra-tumour penetration of spleen cells

G. Lespinats[1], M. Bonnet[1], S. Tlouzeau & C. Burtin[2]

[1]*Institut de Recherches Scientifiques sur le Cancer BP no. 8, 94802 Villejuif Cedex,* [2]*U 203 INSERM, Faculté de Médecine Necker – Enfants Malades, 75730 Paris, France*

In a previous paper (Burtin *et al.*, 1982), we demonstrated that the i.p. daily injection of serotonin to mice carrying methylcholanthrene-induced fibrosarcomas inhibited tumour growth and lengthened survival of mice. We suggested that increased vascular permeability played an essential role, which probably assisted the intratumoral penetration of host immune anti tumour elements.

The effects of serotonin on the blood vessel wall are complex. They include venous constriction, contraction of most arterial and venous smooth muscle, arterior dilation and inhibition of peripheral adrenergic neurotransmission. Thus, in a given vascular bed, the net effect is determined by the balance between the vasoconstrictor and the vasodilator action (Van Nueten, 1982). Because of the pathologic character of tumour vascularization, (Karlsson *et al.*, 1980, Denekamp & Hobson, 1982, Shubik, 1982) the effect of vasoactive drugs on tumours is often different from that on normal tissues. Vasoconstrictors, such as isoprenaline (Mattsson *et al.*, 1982) do not influence tumour blood flow, suggesting that the tumour vascular bed is normally in a state close to maximal dilation (Karlsson *et al.*, 1983; Mattsson, 1980; Mattsson *et al.*, 1982). It was even demonstrated that vasodilators, such as acetylcholine (Young *et al.*, 1983) or papaverine (Karlsson *et al.*, 1983) decrease tumour blood flow.

The activity of serotonin on tumour blood flow and tumour vascular permeability is not known. In order to investigate the mechanism of action of serotonin on tumour growth, the penetration of spleen cells in tumour, spleen and striated muscle was determined with or without serotonin treatment. The possibility of influencing the intratumoral distribution of immune cells would be of great clinical interest. Labelled spleen cells were injected into tumour-bearing mice after administration of serotonin, according to the method described by Salomon *et al.* (1981).

The MC B6-1 fibrosarcoma was originally induced by s.c. injection of 2 mg methylcholanthrene in a female C57 BL/6 mouse, serially transplanted in syngeneic mice, and used between the 20th and the 25th passage. C57BL/6 mice, 6–20 weeks old, received $10^4$ tumour cells s.c., and were used at different times after inoculation. Serotonin (5-hydroxytryptamine and creatinin sulfate, Prolabo) was injected i.p., 1 mg per animal in 0.2 ml medium. Normal spleen cells were labelled with radioactive sodium chromate ($^{51}Cr$, specific activity: $1\,mCi\,ml^{-1}$); these cells, $5–8\times10^6$, containing $1–2\times10^5$ cpm, in 0.1 ml, were injected i.v. to each animal.

Groups of tumour-bearing animals (4–5 per group) received either serotonin or minimal essential medium i.p. One hour later, all groups received labelled spleen cells. One hour later, in a control group and in a serotonin-treated group, tumour, spleen and striated muscle (quadriceps) were removed, individually weighted and counted in a scintillation counter. Four hours after inoculation of spleen cells, another control group and another serotonin-treated group were similarly treated. For each animal, $cpm\,mg^{-1}$ of organ were calculated. Results are expressed as mean $\pm$ s.e. c.p.m. $mg^{-1}$ organ weight and analyzed by the Student's $t$ test.

In spleen and muscle of small and medium-sized tumour bearing mice, 1 h or 4 h after spleen cell injection, serotonin treatment induced either no modification in the radioactivity or a decrease in the spleen and an increase in the muscle (Table I).

In contrast, in the tumour, penetration was increased in both experiments. In the relatively small tumours (mean wt, 251 mg) the increase was detected at 1 h, and was highly significant at 4 h. In the medium-sized tumours (mean wt, 534 mg) the increase was detected only at 4 h.

In order to determine whether penetration was more greatly modified by serotonin in small tumours than in large tumours, we compared the

Correspondence: G. Lespinats
Received 5 April 1984; accepted 27 June 1984.

© The Macmillan Press Ltd., 1984

**Table I** Uptake of $^{51}Cr$ labelled spleen cells in spleen, muscle & tumour of tumour-bearing mice

| *Time after spleen cell injection* | | | *cpm mg$^{-1}$ (mean ± se)* Control | Serotonin | P |
|---|---|---|---|---|---|
| Expt. 1 | 1 h | Spleen | 112±11 | 97±9 | NS |
| | | Muscle | 0.80±0.09 | 1.53±0.16 | <0.01 |
| | | Tumour | 1.35±0.07 | 2.61±0.66 | <0.05 |
| | 4 h | Spleen | 144±11 | 139±11 | NS |
| | | Muscle | 0.77±0.02 | 1.03±0.07 | <0.01 |
| | | Tumour | 1.20±0.07 | 2.09±0.12 | <0.001 |
| Expt. 2 | 1 h | Spleen | 315±22 | 171±40 | <0.02 |
| | | Muscle | 0.48±0.05 | 0.98±0.17 | <0.05 |
| | | Tumour | 1.49±0.15 | 1.25±0.14 | NS |
| | 4 h | Spleen | 391±61 | 393±56 | NS |
| | | Muscle | 0.86±0.06 | 1.16±0.10 | NS |
| | | Tumour | 1.35±0.12 | 2.02±0.28 | <0.05 |

Experiment 1: mean tumour weight (mg) 251±23
Experiment 2: mean tumour weight (mg) 534±61

**Table II** Uptake of $^{51}Cr$ labelled spleen cells, 4 h after injection, in spleen, muscle & tumour, as a function of transplanted tumour size

| | | *cpm mg$^{-1}$ (mean ± se)* Control | Serotonin | P |
|---|---|---|---|---|
| Small tumours | | | | |
| | Spleen | 290±20 | 263±11 | NS |
| | Muscle | 0.93±0.13 | 0.81±0.04 | NS |
| | Tumour | 2.06±0.21 | 4.00±0.80 | <0.05 |
| Large tumours | | | | |
| | Spleen | 48±18 | 31±6 | NS |
| | Muscle | 1.05±0.42 | 0.97±0.06 | NS |
| | Tumour | 0.97±0.17 | 1.44±0.26 | NS |

Small tumours: mean tumour weight (mg) 184±41
Large tumours: mean tumour weight (mg) 2370±131.

**Table III** Relationship between tumour size and uptake of $^{51}Cr$ labelled spleen cells, 4 h after injection

| *Mean tumour weight (mg)* | *% control in serotonin-treated* |
|---|---|
| 184 | 194 |
| 251 | 174 |
| 534 | 150 |
| 2370 | 148 |

penetration in small tumours (mean wt, 184 mg) and in very large tumours (mean weight 2.370 g), 4 h after spleen cell injection.

Table II shows that c.p.m. mg$^{-1}$ of tumour are much more elevated in small tumours, treated or not by serotonin, and that serotonin induced a nearly 2-fold increase in cell penetration in small tumours, and only a non-significant increase in large tumours.

Thus the results as a whole show that the percent increase in cell penetration induced by serotonin treatment, measured 4 h after spleen cell injection, was greater in small tumours than in large ones (Table III).

Because of the complexity of the activity of serotonin on vessel walls and the disorganization of tumour vasculature, the effect of serotonin on tumours could not be anticipated. Indeed, we observed an increase in penetration of spleen cells in tumour, a slight decrease or no effect in muscle; studies showing a state of maximal dilation (Mattsson *et al.*, 1982) and the absence of autoregulation (Suzuki *et al.*, 1981) of tumour vessel walls suggest that passive vascular beds are not responsive, and may be secondarily influenced by the responding somatic vessels connecting with tumour vessels (Suzuki *et al.*, 1981). The method used here does not allow us to determine whether the mechanism of action of serotonin is direct or indirect, but does illustrate the final result of increased penetration of lymphoid cells inside the tumour.

The well known phenomenon of preferential homing of lymphocytes in the spleen (Ford, 1975) was observed since radioactivity per mg of organ was 100–300 times more elevated than in the tumour and the muscle; however, serotonin did not increase this penetration. Penetration was lower in the muscle than in the tumour and the elevation induced by serotonin in the former tissue was inconsistent. By contrast, the serotonin effect was important in small tumours, where it induced a nearly 2-fold increase in spleen cell penetration, which decreased progressively with tumour growth. It is known that, with increasing size, the proportion of effective vascularization decreases (Denekamp & Hobson, 1982) and the plasma volume is relatively reduced (Karlsson *et al.*, 1980). However, the effect of serotonin remains significant on tumours of 534 mg, grafted 20 days before testing.

In previously described experiments (Burtin *et al.*, 1982) we observed that serotonin treatment reduced tumour growth. This antitumour effect of serotonin was observed even when treatment was begun in mice bearing medium size tumours. Histological examination showed the presence of bands and foci of necrotic and haemorrhagic tissue in the tumours of mice treated with serotonin.

Serotonin is the most important agent for increasing vascular permeability in mice (Schartz *et al.*, 1977). It plays an important role in the migration of lymphocytes to the site of delayed-type hypersensitivity (Gershon *et al.*, 1975; Askenase *et al.*, 1982). It has been suggested that vasoactive amines are important in the recruitment of inflammatory cells into tumours (Lynch & Salmon, 1977). It could increase, within the tumour, infiltrating elements with an antitumour activity (Klein *et al.*, 1980).

However, the vascular effect of serotonin is perhaps not sufficient to explain its antitumoral activity. *In vitro* experiments showed that mast cells were cytotoxic to mouse fibrosarcoma cells. Reserpine blocked tumour killing, suggesting serotonin as the principal agent of tumour cell killing by mast cells. (Farram & Nelson, 1980).

The increase in mast cell number demonstrated in tissues of tumour bearing mice (Galoppin *et al.*, 1984) could play an important role in antitumour defense, acting by more than one mechanism.

**Acknowledgements**

We thank V. Lascaux for excellent technical assistance.

### References

ASKENASE, P.W., METZLER, C.M. & GERSHON, R.K. (1982). Localization of leucocytes in sites of delayed-type hypersentivity and in lymph modes: dependence on vasoactive amines. *Immunology*, **47**, 239.

BURTIN, C., SCHEINMANN, P., SALOMON, J.C., LESPINATS, G. & CANU, P. (1982). Decrease in tumour growth by injections of histamine or serotonin in fibrosarcoma-bearing mice: influence of H1 and H2 histamine receptors-*Br. J. Cancer*, **45**, 54.

DENEKAMP, J. & HOBSON, B. (1982). Endothelial cell proliferation in experimental tumours-*Br. J. Cancer*, **46**, 711.

FARRAM, E. & NELSON, D.S. (1980). Mouse mast cells as anti-tumor effector cells. *Cell Immunol.*, **55**, 294.

FORD, W.L. (1975). Lymphocyte migration and immune responses. *Prog. Allergy*, **19**, 1.

GALOPPIN, L., RAYNAUD, F., PONVERT, C. & 5 others. (1984). Tissue histamine levels and mast cell number in tumour bearing mice. *Agents Actions*, **14**, 494.

GERSHON, R.K., ASKENASE, P.W. & GERSHON, M.D. (1975). Requirement for vasoactive amines for production of delayed-type hypersensitivity skin reactions. *J. Exp. Med.*, **142**, 732.

KARLSSON, L., ALPSTEN, M., APPELGREN, K.L. & PETERSON, H.I. (1980). Intratumor distribution of blood flow and of vascular volume in a transplantable rat fibrosarcoma. *J. Cancer Res. Clin. Oncol.*, **98**, 213.

KARLSSON, L., ALPSTEN, A., MATTSSON, J. & PETERSON, H.I. (1983). Influence of vasoactive drugs on the intratumor distribution of blood flow and vascular volume in a transplantable rat fibrosarcoma. *J. Cancer Res. Clin. Oncol.*, **105**, 212.

KLEIN, E., VANKY, F., GALILI, U., VOSE, B.M. & FOPP, M. (1980). Separation and characteristics of tumour-infiltrating lymphocytes in man – *Contemp. Topics Immunobiol.*, **10**, 79.

LYNCH, N.R. & SALOMON, J.C. (1977). Passive local anaphylaxis: demonstration of antitumor activity and complementation of intratumor BCG. *J. Natl. Cancer Inst.*, **58**, 1093.

MATTSSON, J., ALPSTEN, M., APPELGREN, L. & PETERSON, H.I. (1980). Influence of Noradrenaline on local tumour blood flow. *Eur. J. Cancer*, **16**, 99.

MATTSSON, J., LILJA, J. & PETERSON, H.I. (1982). Influence of vasoactive drugs on local tumour blood flow. *Eur. J. Cancer*, **18**, 677.

SALOMON, J.C., LYNCH, M.R., GHEORGHIU, M., GALINHA, A., LASCAUX, V. & PRIN, J. (1981). Resistance factors to intralesional immunotherapy with BCG or Coryne bacterium Parvum in rat tumor. *Cancer Immunol. Immunoth.*, **10**, 87.

SCHWARTZ, A., ASKENASE, P.W. & GERSHON, R.K. (1977). The effect of locally injected vasoactive amines on the elicitation of delayed-type hypersensitivity. *J. Immunol.*, **118**, 159.

SHUBIK, P. (1982). Vascularization of tumor: a review. *J. Cancer Res. Clin. Oncol.*, **103**, 211.

SUZUKI, M., HORI, K., ABE, I., SAITO, S. & SATO, H. (1981). A new approach to cancer chemotherapy: selective enhancement of tumor blood flow with angiotensin II. *J. Natl Cancer Inst.*, **67**, 663.

VAN NUETEN, J.M. (1983). 5-Hydroxytryptamine and precapillary vessels. *Fed. Proc.*, **42**, 223.

YOUNG, S.W., MULLER, H.H. & MARINCEK, B. (1983). Response of neoplastic and normal vasculature to acetylcholine. *Eur. J. Cancer Clin. Oncol.*, **19**, 383.